# Editorial 15th Anniversary of Pharmaceutics—Improvement of Drug Bioavailability

**DOI:** 10.3390/pharmaceutics16121568

**Published:** 2024-12-08

**Authors:** Thierry F. Vandamme

**Affiliations:** Centre de Recherche en Biomédecine de Strasbourg (CRBS), University of Strasbourg, 1 rue Eugène Boeckel, 67000 Strasbourg, France; vandamme@unistra.fr

## 1. Introduction

Improving the health of humans and animals depends on the discovery of new active molecules as well as improving the bioavailability of molecules already marketed. Improving drug bioavailability is crucial for advancing medicine, optimizing treatment regimens, and making therapies more effective, safer, and affordable for patients globally. Research on improving the bioavailability of drugs is highly important for all of these reasons, each with significant implications for both patient care and the pharmaceutical industry. In this field, academic and industrial research is an extremely dynamic sector. Its ingenuity can be observed in many aspects that researchers have focused on in recent decades.

Drugs with low bioavailability often require higher doses to achieve the desired therapeutic effect. Improving bioavailability allows for lower doses to be used, which can lead to more effective treatment with fewer side effects. Also, with better bioavailability, medications can act more consistently and predictably, leading to more reliable treatment outcomes [1,2,3]. High doses of a drug often increase the risk of side effects and toxicity, especially for drugs that have narrow therapeutic windows. By improving bioavailability, lower doses can be effective, reducing the likelihood of adverse reactions and improving patient safety [4]. Enhanced bioavailability can lead to reduced dosage requirements, which in turn can decrease the cost of treatment. This aspect is particularly crucial in drugs that are expensive to manufacture, such as biologics. Better bioavailability also translates to fewer doses needed over time, which can improve patient adherence and reduce healthcare costs [5]. Many patients prefer oral drugs for convenience, but certain drugs have poor bioavailability when taken orally. Improving bioavailability can make more drugs suitable for oral administration, which is generally preferred over injections or infusions. Better bioavailability can also mean fewer doses or less frequent dosing, making it easier for patients to follow their treatment regimens [6]. The body’s natural barriers, such as the intestinal lining and blood–brain barrier, pose challenges for drug delivery and, in particular, the possibility to cross them. Bioavailability research aims to find ways to navigate these barriers, which is essential for delivering therapeutic agents to specific tissues, including hard-to-reach areas like the brain [7,8]. A large percentage of new drugs are poorly water-soluble or poorly permeable, making them challenging to deliver effectively. Research in bioavailability enhancement provides pharmaceutical companies with more tools and strategies to turn promising drug candidates into viable treatments. Innovations in bioavailability could also pave the way for new drug types, such as gene therapies, that require unique delivery approaches [9]. Improved bioavailability can make essential drugs more accessible and effective in low-resource settings, where cold storage or intravenous administration might not be feasible. Enhancing oral bioavailability, for example, makes drugs more accessible in regions lacking advanced healthcare infrastructure [10].

Scientific literature as well as patents describe a large number of strategies and technologies that have been developed to improve the bioavailability of active pharmaceutical ingredients (APIs). Improving the bioavailability of drugs, or the fraction of an administered dose that reaches systemic circulation, is essential for maximizing therapeutic effects while minimizing dose requirements and side effects. Key strategies used to enhance bioavailability have largely been developed. From the formulation strategies, the use of nanotechnology (nanoparticles, liposomes, and nano-emulsions) can improve drug solubility [11], stability, and cellular uptake, enhancing absorption and bioavailability. Micronization can also improve bioavailability by reducing drug particle size to the micrometer or nanometer scale, which increases surface area, promoting faster dissolution and absorption. Solid dispersions, by dispersing drugs in polymers, can improve solubility and enhance dissolution rates, especially for poorly water-soluble drugs. Prodrugs can be chemically modified into a more bioavailable form and can be converted back to their active form once they enter systemic circulation. Cyclodextrins and inclusion complexes can form new entities with poorly soluble drugs, increasing their solubility without changing their molecular structure. For polymeric micelles, drugs can be loaded into polymer-based micelles, enhancing solubility and increasing absorption. Self-Emulsifying Drug Delivery Systems (SEDDS) are able to form fine emulsions in the GI tract, promoting absorption for poorly water-soluble drugs. Salts and ester forms have the intrinsic ability to change a drug to a salt or ester form, which can improve its solubility, stability, and permeability. PEGylation, i.e., adding polyethylene glycol (PEG) chains to a drug, can protect it from enzymatic degradation, improving bioavailability, particularly for peptide drugs.

Permeation enhancers are composed of some agents that are able to temporarily disrupt cell membranes or tight junctions in the intestinal wall, increasing drug permeability. Examples of developments include fatty acids, surfactants, and bile salts [12]. Lipid-based carriers like micelles, phospholipids, and certain emulsions can also facilitate transport across biological membranes. Formulations with bioadhesive agents prolong the residence time of the drug at the absorption site and therefore enhance absorption. Controlled-release and targeted delivery formulations are designed to release drugs slowly over time, allowing for more consistent blood levels, reducing degradation before reaching target tissues, and enhancing bioavailability [13]. Use of antibodies, peptides, or other ligands for targeted drug delivery to specific tissues or cells helps ensure that more of the drug reaches its target, improving overall bioavailability. The mucoadhesive formulations are able to adhere to the mucosal lining of the GI tract, allowing drugs to stay in contact with absorption sites longer. By transdermal and intranasal delivery, the routes bypass first-pass metabolism, increasing the bioavailability of certain drugs. By using the strategies of cytochrome P450 inhibitors, some drugs are rapidly metabolized by the liver before reaching systemic circulation. Using enzyme inhibitors can slow this metabolism, allowing more of the drug to be absorbed [14]. Also, efflux pump inhibitors, such as P-glycoprotein (P-gp), actively pump drugs out of cells. Co-administration with P-gp inhibitors can reduce drug efflux and increase absorption [15]. Physiological modulation can also improve the bioavailability of drugs. Gastrointestinal pH affects drug solubility and absorption. For instance, acidic drugs may be better absorbed in a low pH environment, so co-administering pH modifiers may enhance bioavailability. Food interactions can be used as an alternative means. Certain foods can increase the absorption of drugs, often by enhancing solubility or slowing gastric emptying, which can prolong drug residence time in the intestines. For drugs with poor oral bioavailability, alternative routes such as sublingual, buccal, transdermal, or intranasal administration can bypass first-pass metabolism and increase systemic availability. Combining these strategies can often yield even better results. However, the choice of strategy must be tailored to the specific drug’s properties, its therapeutic goal, and the patient’s needs. The selection of an appropriate strategy depends on the drug’s characteristics, the intended route of administration, and the therapeutic goals. Combining these approaches often yields improved results, providing better therapeutic outcomes and patient compliance.

## 2. An Overview of Published Articles

This anniversary Special Issue for the topic “Improvement of bioavailability” has collected manuscripts with original strategies that pharmaceutical researchers and scientists employ to enhance the bioavailability of drugs. In order to expand the therapeutic utility of human DNase I, Stamm et al. (contribution 1) developed a variant of the enzyme that has both a prolonged systemic half-life and a higher catalytic activity compared to Dornase alfa (Pulmozyme^®^), the recombinant form of DNase I approved for inhaled cystic fibrosis therapy.

Patil et al. (contribution 2) investigate the application of nanostructured lipid carriers (NLCs) to enhance the bioavailability of Apixaban. NLCs were prepared using the high-pressure homogenization method. The influence of independent variables, the amount of Tween 80, HPH pressure, and the number of HPH cycles were studied using a 23-factorial design. In the case of the thermal and crystallographic properties of the drug, the NLCs showed drug entrapment without altering its potency. The in vitro drug release studies revealed an immediate release pattern, followed by sustained release for up to 48 h. In vivo pharmacokinetic experiments demonstrated that Apixaban-loaded NLCs exhibited higher values of t_1/2_ compared to free drugs, indicating improved bioavailability. Moreover, a decrease in the elimination rate constant (Kel) reflected the sustained effect of Apixaban with NLCs. NLCs offer improved oral absorption rates and enhanced therapeutic impact compared to free drugs, potentially reducing dose frequency and improving patient outcomes.

Based on an original concept, Jamshaid et al. (contribution 3) developed a new type of polymer-free hydrogel made only from nonionic surfactants, oil, and water. Such a system is produced by taking advantage of the physicochemical behavior and interactions between nonionic surfactants and oil and water phases, according to a process close to spontaneous emulsification used in the production of nano-emulsions. Beyond the originality of the concept, these nanoemulgels appear as very promising systems able to encapsulate and deliver various molecules with different solubilities. In the first section, the authors propose a comprehensive investigation of the gel formation process and its limits through oscillatory rheological characterization, the characterization of the sol/gel transitions, and gel strength. The second section is focused on the follow-up of the release of an encapsulated model hydrophilic molecule and the impact of the rheological gel properties on the release profiles.

Furuishi et al. (contribution 4) synthesized a family of novel ionic liquids (ILs) with meglumine (MGM) as the cation and tartaric acid (TA), azelaic acid (AA), geranic acid (GA), and capric acid (CPA) as anions, using pharmaceutical additives via simple acid–base neutralization reactions. The authors investigated the solubilization of 15 drugs with varying pKa and partition coefficient (log P) values using these ILs and performed a comparative analysis. Furthermore, Furuishi et al. present MGM-IL as a new skin permeation enhancer for the drug model flurbiprofen (FRP). They confirmed that AA/MGM-IL improves the skin permeation of FRP through hairless mouse skin. Moreover, AA/MGM-IL enhanced drug skin permeability by affecting keratin rather than stratum corneum lipids, as confirmed by ATRFTIR. From the obtained results, they conclude that MGM-ILs exhibited potential as drug solubilizers and skin permeation enhancers of drugs.

In the review of the transdermal delivery of non-steroidal anti-inflammatory drugs (NSAIDs), Balmanno et al. (contribution 5) described various strategies used to increase the solubility of poorly soluble NSAIDs and enhance their permeation through skin, such as the modification of the vehicle, the modification of or bypassing the barrier function of the skin, and using advanced nano-sized formulations. Furthermore, the authors focused their review on a simple yet highly versatile microemulsion system that has been found to be a cost-effective and highly successful technology to deliver poorly water-soluble NSAIDs.

## 3. Conclusions

The future of improving drug bioavailability will be shaped by a multidisciplinary approach, combining biotechnology, materials science, personalized medicine, and AI. Together, these advancements are likely to produce therapies that are more effective, safer, and increasingly tailored to individual patient needs, revolutionizing how drugs work in the body. Three-dimensional printing enables the customization of drug dosages, release profiles, and shapes to suit individual patient needs. It will also allow the development of complex structures, such as multilayer tablets that release drugs at different rates, which can optimize bioavailability. In the future, pharmacies or even hospitals could print medications tailored to patients’ genetic profiles, adjusting for factors like enzyme activity that influence bioavailability. Recent research shows that the gut microbiome can influence drug metabolism and bioavailability. Understanding and possibly manipulating the gut microbiome will help optimize drug absorption for individual patients, especially for orally administered medications. Finally, we can hope that in the near future, AI and machine learning algorithms will aid in drug design by predicting how chemical modifications may impact bioavailability, identifying optimal formulations, and personalizing doses based on a patient’s physiology and genetics. This approach will accelerate the development of more bioavailable drugs while reducing the costs associated with trial and error in formulation development.
